# Comparison between accuracy of augmented reality computed tomography-based and portable augmented reality-based navigation systems for cup insertion in total hip arthroplasty

**DOI:** 10.1038/s41598-024-59115-2

**Published:** 2024-04-09

**Authors:** Masahiro Hasegawa, Yohei Naito, Shine Tone, Akihiro Sudo

**Affiliations:** Department of Orthopaedic Surgery, Mie University Graduate School of Medicine, 2-174 Edobashi, Tsu City, Mie 514-8507 Japan

**Keywords:** Medical research, Rheumatology

## Abstract

Augmented reality (AR) has been used for navigation during total hip arthroplasty (THA). AR computed tomography (CT)-based navigation systems and AR-based portable navigation systems that use smartphones can also be used. This study compared the accuracy of cup insertion during THA using AR-CT-based and portable AR-based navigation systems. Patients with symptomatic hip disease who underwent primary THA in the supine position using both AR CT-based and portable AR-based navigation systems simultaneously between October 2021 and July 2023 were included. The primary outcome of this study was the absolute difference between cup angles in the intraoperative navigation record and those measured on postoperative CT. The secondary outcome was to determine the factors affecting the absolute value of the navigation error in radiographic inclination (RI) and radiographic anteversion (RA) of the cup, including sex, age, body mass index, left or right side, approach, and preoperative pelvic tilt. This study included 94 consecutive patients. There were 11 men and 83 women, with a mean age of 68 years. The mean absolute errors of RI were 2.7° ± 2.0° in the AR CT-based and 3.3° ± 2.4° in the portable AR-based navigation system. The mean absolute errors of RA were 2.5° ± 2.1° in the AR CT-based navigation system and 2.3° ± 2.2° in the portable AR-based navigation system. No significant differences were observed in RI or RA of the cup between the two navigation systems (RI: p = 0.706; RA: p = 0.329). No significant factors affected the absolute value of the navigation errors in RI and RA. In conclusion, there were no differences in the accuracy of cup insertion between the AR CT-based and portable AR-based navigation systems.

## Introduction

Computer navigation is used in total hip arthroplasty (THA) to accurately place acetabular cups. Accurate cup placement has been associated with the prevention of impingement, dislocation, and revision^1–3^. While navigation may improve longevity after THA^1,3^, its success and, therefore, universal acceptance remain contentious^4^.

Augmented reality (AR) is a technology that overlays three-dimensional (3D) computer graphic images into the view of the real world^5,6^. Medical AR is an extension of computer-assisted surgery with various applications, including direct visualization of 3D radiological images directly on the patient and intraoperative guidance using preoperative plans^5,6^. No discernible differences in the accuracy of cup placement were observed between AR-trained groups and those trained by an expert surgeon^7^. Recently, AR technology has been clinically used for THA. CT-based navigation with these AR technologies was designated AR CT-based navigation in this study. Currently, Holonavi One (Holonavi Medical Technology Inc., Ichinomiya, Japan)^8^ is used AR CT-based navigation during THA. Surgeons can use AR computed tomography (CT)-based navigation systems (Holonavi One)^8^ and portable AR-based navigation systems using smartphones (AR-Hip; Zimmer Biomet Japan, Tokyo, Japan)^9,10^. One of the advantages of the AR CT-based navigation system is its ease of use without an AR headset, which may be difficult to use with a surgical helmet^8^. The AR CT-based navigation system enables surgeons to enhance cup placement accuracy in a supine position, compared with freehand placement^8^. In addition, surgeons can confirm not only the bone but also the muscles and vessels on the monitor of the AR CT-based navigation system^8^. Both systems demonstrate accurate cup insertion^8–13^.

In the present study, both AR CT-based and portable AR-based navigation systems were used simultaneously, and we compared the accuracy of cup insertion in THA using both systems. We hypothesized that AR CT-based and portable AR-based navigation systems would provide similar accuracy in acetabular cup placement.

## Materials and methods

### Patients

The inclusion criteria were patients with symptomatic hip disease who underwent primary cementless THA in the supine position under general anesthesia using both the AR CT-based and portable AR-based navigation systems simultaneously between October 2021 and July 2023. The exclusion criteria were hips that underwent THA via a posterior approach, those with high dislocation, or those requiring subtrochanteric osteotomy. The same surgeon (M.H.) performed all procedures. The hip was exposed using a modified Watson–Jones approach (anterolateral supine approach; ALS) or direct anterior approach (DAA) on a traction table. The DAA was selected when a traction table was available. The ALS was preferred in instances when the table was unavailable or in cases of high dislocation of the hip (Crowe groups II and III)^14^ or excessive anteversion (> 35°) of the femoral neck.

CT was performed from the pelvis to the knee joint preoperatively. CT scans with metal artifacts were acquired using the following scanner settings: 120 kV and 150 mA; slice thickness, 2.0 mm; pixel resolution, 512 × 512. ZedHip required a slice thickness of less than 2.0 mm for accurate measurements. RI and RA were planned at 40º and 15º relative to the functional pelvic plane (FPP), respectively. The FPP was established by adjusting the anterior pelvic plane (APP) posteriorly or anteriorly in the sagittal plane until it aligned parallel to the preoperative CT table^15,16^. Implant sizes were preoperatively determined for all cases using a 3D digital templating system (ZedHip; LEXI Co., Tokyo, Japan).

### Surgical technique

**Figure 1:**
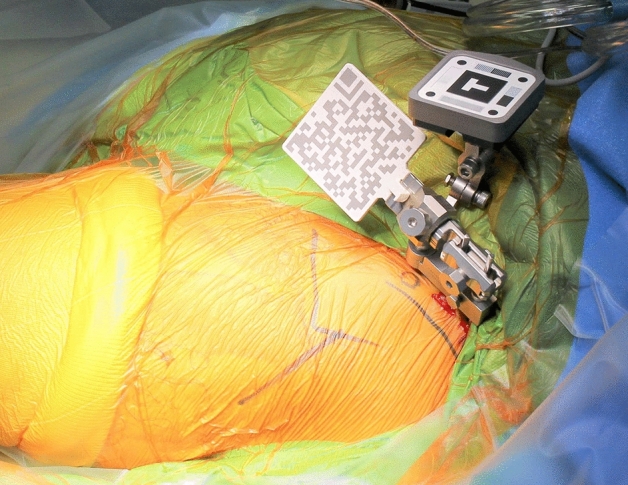
Augmented reality (AR) markers for computed tomography (CT)-based and portable navigation systems are attached to the two pins inserted into the pelvis.

**Figure 2:**
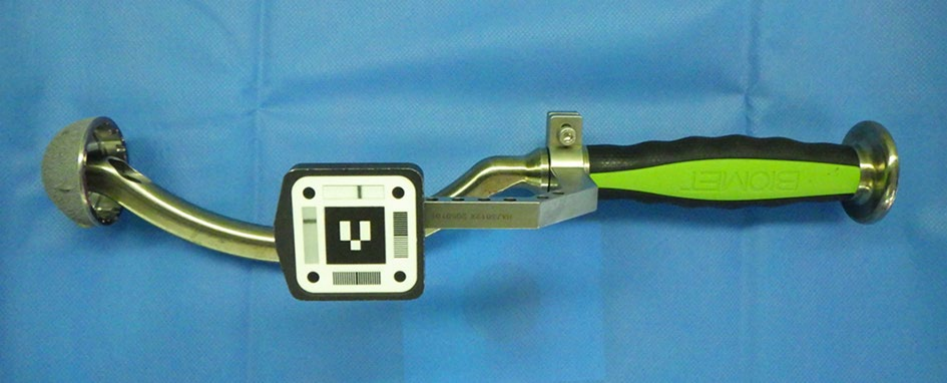
Photograph of the standard cup holder with the augmented reality (AR) marker.

**Figure 3:**
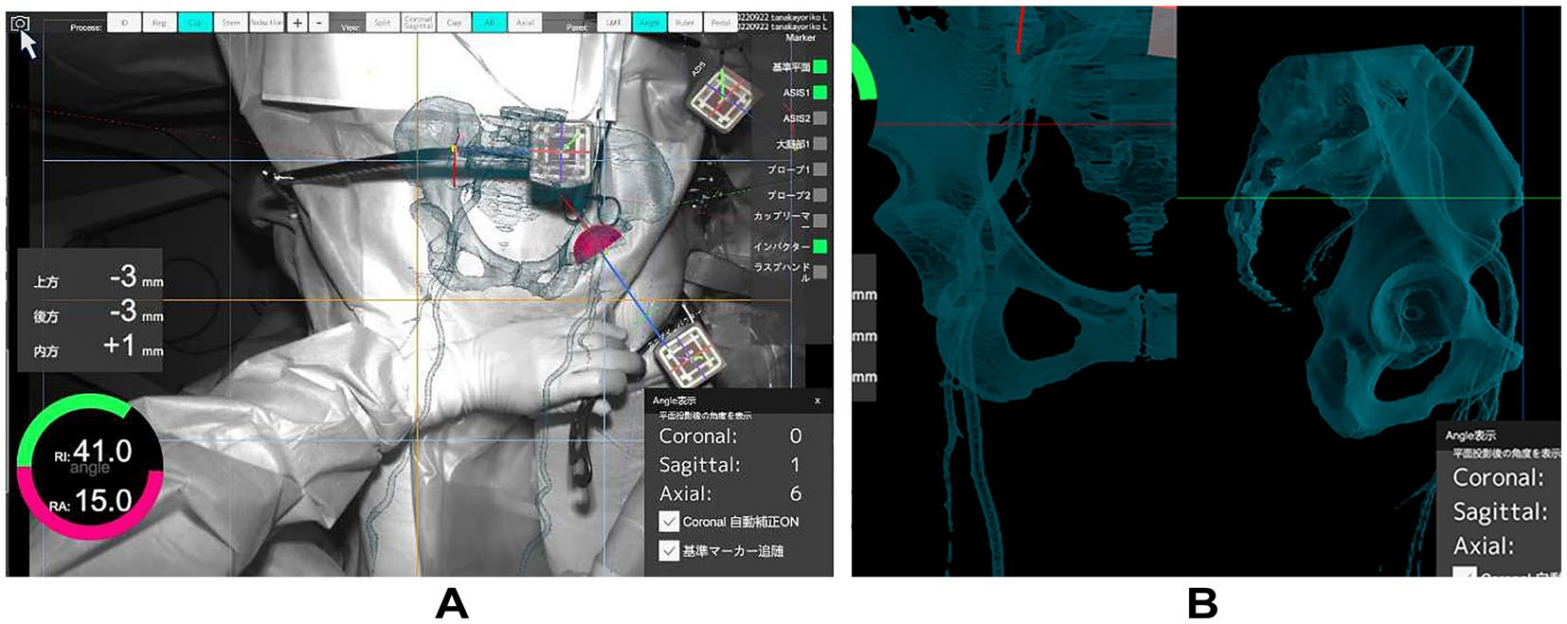
Screens of the augmented reality (AR) computed tomography (CT)-based navigation system are shown. (**A**) Surgeons can view a three-dimensional model of the pelvis on the real surgical field on the monitor. Radiographic inclination (RI) and radiographic anteversion (RA) of the cup are provided in real time. (**B**) Vessel locations can be displayed during surgery.

**Figure 4:**
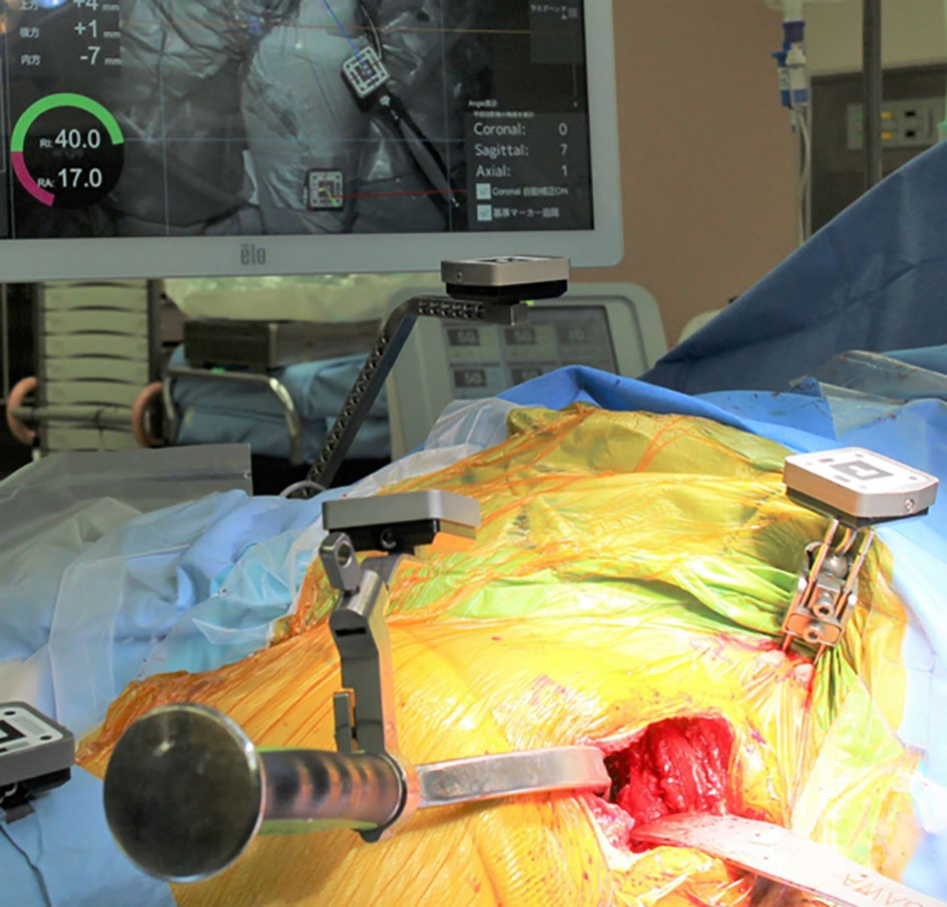
Cup insertion process using the augmented reality (AR) computed tomography (CT)-based navigation system.

**Figure 5:**
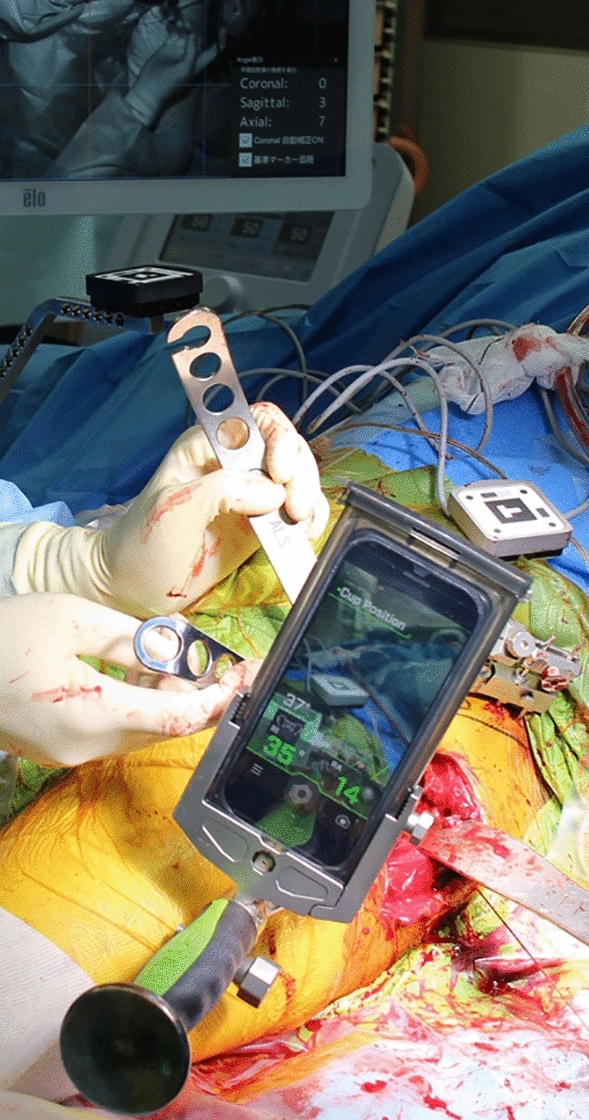
In the portable augmented reality (AR)-based navigation system, the smartphone is attached to the cup impactor. The smartphone recognizes the AR marker attached to the fixation pins at the pelvis. The display of the smartphone shows the radiographic inclination (RI) and radiographic anteversion (RA) of the cup.

Two pins with a diameter of 3.2 mm were inserted into the iliac crest in parallel through small incisions after draping. The AR marker for the CT-based navigation system was connected to the pins with clamps. Another AR marker for the portable navigation system was fixed to the same pin (Fig. 1). The bilateral anterior superior iliac spines (ASIS) and pubic tubercles were registered, and the APP was identified. However, registration of the pubic tubercle has no effect on cup orientation in either navigation system, because percutaneous palpation of the pubis is known to be imprecise^16^. The FPP was determined according to preoperative CT and bilateral ASIS registration in the AR CT-based navigation system. Gravitational vectors can be calculated from the gyro sensor built into a smartphone in the portable AR-based navigation system^9,10^, and the FPP can be determined. In the AR CT-based navigation system, initial paired-point matching and surface matching were performed by digitizing 28 points in the acetabulum. Both AR CT-based and portable AR-based navigation systems were used simultaneously. The AR marker was attached to the standard cup holder using a screw (Fig. 2) and the cup was inserted using the AR-CT-based navigation system (Figs. 3, 4). After 1-mm underreaming, press-fit fixation was obtained without screws in all cases. A G7 PPS Finned BoneMaster Limited hole shell (Zimmer Biomet, Warsaw, IN, USA) was used. After recording the RI and RA values displayed on the monitor of the AR CT-based navigation system (Fig. 4), the AR marker attached to the cup holder was removed. Subsequently, a smartphone was attached to the cup holder and scanned the QR code connected to the side of the pelvic pins. The RI and RA values were then displayed on the smartphone and recorded (Fig. 5). The cup position remained unchanged throughout this procedure in all cases. The final cup angles were verified using a portable AR-based navigation system (Fig. 5). Using postoperative CT, the accuracy of cup angles was compared between the two AR navigation systems.

### Outcome measurements

**Figure 6:**
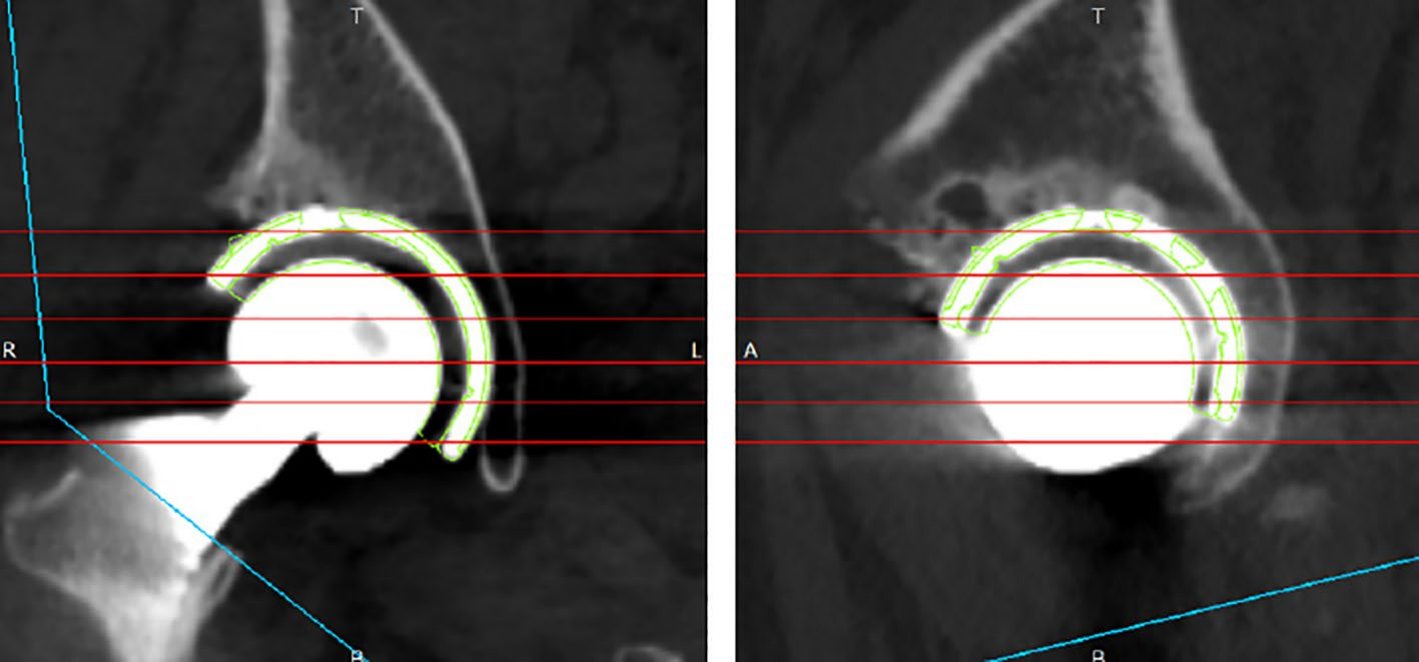
Measurement of radiographic inclination (RI) and radiographic anteversion (RA) using the 3-dimensional digital templating system (ZedHip).

Radiographic RI and RA were measured with respect to the FPP using CT performed from the pelvis to the knee joint 2 weeks postoperatively and the 3D digital templating system (ZedHip, Fig. 6) by one observer (Y.N.). The reliability of intra-observer and inter-observer for this measurement has been evaluated previously^14^. The absolute target errors in RI and RA were defined as the differences between the preoperative target angles and the angles measured on postoperative CT. Intraoperative RI and RA were recorded using both navigations. We defined absolute navigation errors in RI and RA as the absolute difference between the angles in the navigation records and postoperative CT measurements^17^. The absolute errors between the preoperative target angles and the angles recorded in the AR CT-based navigation system were also assessed. The primary outcome was the absolute navigation errors in both the AR CT-based and portable AR-based navigation systems. The secondary outcome was to determine the factors affecting the absolute value of the navigation error in RI and RA, including sex, age, body mass index, left or right side, approach, and preoperative pelvic tilt.

This study was approved by the Institutional Review Board of Mie University (H2018-083), and all patients provided written informed consent prior to participation. All procedures were performed in accordance with the principles of the 1964 Declaration of Helsinki.

### Statistical analysis

In previous studies^8,10^, the difference in RI errors between AR CT-based and portable AR-based navigation systems was 0.6°. Based on this finding, a total sample size of 74 hips was required to detect a significant difference between the groups (ɑ = 0.05, power = 0.8).

The Wilcoxon signed-rank test was used to compare the accuracy of cup angles between the AR navigation systems. Multiple regression analysis was conducted to determine the factors affecting the absolute value of the navigation error in RI and RA, including sex, age, body mass index, left or right side, approach, and preoperative pelvic tilt. Correlation analyses were performed between the errors of the AR-based navigation systems in the RI and RA. P-values of < 0.05 were considered statistically significant, using EZR (Jichi Medical University, Shimotsuke, Japan) version 1.61^18^.

## Results

This study included 94 consecutive patients. ALS was used in 52 hips and DAA in the remaining 42 hips. There were 11 men and 83 women, with a mean age of 68 years (range 35–90 years) and a mean body mass index of 24.9 kg/m^2^ (range 16.3–37.0 kg/m^2^). The preoperative diagnosis was osteoarthritis in 91 patients and idiopathic osteonecrosis of the femoral head in 3 patients.

The mean absolute target errors were 3.1° ± 2.4° in RI and 3.2° ± 2.3° in RA. The mean absolute navigation errors of RI were 2.7° ± 2.0° in the CT-based navigation system and 3.3° ± 2.4° in the portable navigation system. The mean absolute navigation errors of RA were 2.5° ± 2.1° in the AR CT-based navigation system and 2.3° ± 2.2° in the portable AR-based navigation system (Table 1). RI or RA of the cup showed no significant differences between the two navigation systems (RI: p = 0.706; RA: p = 0.329). The absolute errors between the preoperative target angles and the angles in the AR CT-based navigation records for RI and RA were 1.7° ± 1.3° and 2.1° ± 2.0°, respectively.

**Table 1:** Accuracy of navigation systems in supine position.

Navigation	Authors	Company	Absolute value of error^a^		Absolute value of navigation error^b^	
			Inclination (°)	Anteversion (°)	Inclination (°)	Anteversion (°)
CT-based	Kalteis et al.^19^	Brainlab	4.2 ± 4.0	5.3 ± 5.3	3.0 ± 2.6	3.3 ± 2.3
	Matsuki et al.^20^	Stryker	2.8 ± 2.5	2.8 ± 1.9		
	Hasegawa et al.^8^	Holonavi Medical Technology	2.8 ± 2.2	2.9 ± 2.3	2.5 ± 1.7	2.5 ± 2.2
	Present study	Holonavi Medical Technology	3.1 ± 2.4	3.2 ± 2.3	2.7 ± 2.0	2.5 ± 2.1
Image-free	Tsukada and Wakui^22^	B. Braun Aesculap	2.8 ± 2.5	4.2 ± 3.0	2.4 ± 2.0	3.7 ± 2.3
	Fukunishi et al.^21^	B. Braun Aesculap			3.0 ± 2.6	5.0 ± 3.5
	Kalteis et al.^19^	Brainlab	3.6 ± 4.0	4.2 ± 5.5	2.9 ± 2.2	4.2 ± 3.3
Portable	Kamenaga et al.^25^	OrthAlign			2.6 ± 2.7	2.8 ± 2.7
	Takada et al.^26^	OrthAlign	3.3 ± 2.7	3.8 ± 3.4		
	Hasegawa et al.^17^	OrthAlign	3.8 ± 2.7	3.3 ± 2.5	3.7 ± 2.8	3.0 ± 2.6
	Hayashi et al.^24^	OrthAlign	2.6 ± 1.9	2.7 ± 2.2	2.7 ± 2.1	2.7 ± 1.8
	Tetsunaga et al.^27^	OrthAlign			3.3 ± 2.4	3.4 ± 2.2
	Hasegawa et al.^23^	Naviswiss AG	4.1 ± 3.2	4.3 ± 3.2	2.8 ± 2.2	2.8 ± 2.0
	Present study	Zimmer Biomet	2.8 ± 3.0	2.9 ± 2.0	3.3 ± 2.4	2.3 ± 2.2

Absolute errors are given as means ± standard deviation.

^a^Absolute deviation of the postoperative measured angle from the target position.

^b^Absolute difference between the navigation recorded and the postoperative measured angle.

The percentages of hips with RI errors of > 5° were 9% and 13% for the AR CT-based and portable AR-based navigation systems, respectively. The percentages of hips with RA errors of > 5° were 14% and 7% for the AR CT-based and portable AR-based navigation systems, respectively. No hips showed navigation errors of > 10° in the AR CT-based navigation group for RI and RA. The percentages of hips with RI and RA errors of > 10° were 3% and 1%, respectively.

Multiple regression analysis demonstrated no significant factors affecting the absolute value of the navigation error in RI and RA (Table 2). Weak correlations were observed between the errors of both AR-based navigation systems (RI: r = 0.284, p = 0.006; RA: r = 0.412, p < 0.001).

**Table 2:** Multivariate analysis of factors affected the absolute value of navigation error in RI and RA.

	AR CT-based navigation system		Portable AR-based navigation system	
	RI	RA	RI	RA
Sex	0.516	0.056	0.866	0.914
Age	0.584	0.275	0.225	0.076
Body mass index	0.793	0.879	0.374	0.481
Side	0.146	0.053	0.099	0.376
Approach	0.45	0.076	0.704	0.073
Preoperative pelvic tilt	0.700	0.789	0.785	0.824

All values presented the p value of multivariate analysis.

*RI* radiographic inclination, *RA* radiographic anteversion, *AR* augmented reality, *CT* computed tomography.

## Discussion

The most important finding of the present study was that cup placement accuracies were equivalent between the two AR navigation systems. The simultaneous use of both AR CT-based and portable AR-based navigation systems was a strong point of the present study in evaluating accuracy.

The advantages of the portable AR-based navigation system are that it does not require preoperative CT with radiation exposure and is inexpensive, especially for nonheavy users. Defining the acetabular component angle alone may be insufficient. To control cup inclination and anteversion, surgeons might trust both AR CT-based and AR-portable navigation systems. However, AR CT-based navigation systems have many options, including reamer position, cup position, pelvic movements, and muscles^8^. In addition, vessels can be depicted three-dimensionally using an AR CT-based navigation system, and surgeons can safely insert screws without causing vascular injury, even in complex revisions (Fig. 3B)^8^. Registration of the muscles and vessels was not performed during surgery, and their visualization depended mainly on the positioning of the patient. The potential benefits of identifying blood vessels were not demonstrated, as no screws were utilized in this study.

Ogawa et al.^10^, who developed the portable AR-based navigation system (AR-Hip), reported on the results of this system, and errors evaluated using postoperative CT were 1.9° ± 1.3° and 2.8° ± 2.2° in radiographic inclination (RI) and radiographic anteversion (RA), respectively. The errors using AR CT-based navigation system were 2.5° ± 1.7° for RI and 2.5° ± 2.2° for RA^8^. Table 1 summarizes the results of previous studies on the accuracy of navigation systems in the supine position. The navigation errors using CT-based navigation were 2.5°–3.0° for RI and 2.5°–3.3° for RA^8,19,20^. For image-free navigation, accurate registration of the APP is required. Percutaneous palpation of the pubic tubercle is quite imprecise^16^, and the thickness of the soft tissue overlying the pubis affects anteversion accuracy. The navigation errors using image-free navigation are 2.4°–3.0° for RI and 3.7°–5.0° for RA^19,21,22^. Using portable navigation in the supine position, the absolute values of the navigation error in RI reportedly range 2.6°–3.7°, and RA errors range 2.7°–3.4°^17,23–27^. The radiographic RA does not affect the registration of the pubic symphysis in portable navigation systems. This could contribute to the improved accuracy of RA in portable navigation. The accuracy of cup angle using AR is comparable to that previously reported for CT-based navigation^8,19,20^ and portable navigation systems^17,23–27^. CT-based navigation with AR technologies enables surgeons to recognize not only bone but also soft tissue, whereas portable AR-based navigation is limited to navigating bone only. In this study, we observed that the RA tended to be less accurate in the AR CT-based navigation, while the RI showed the opposite trend. The reduced accuracy of RA in this system could derive from changes in pelvic tilt between the preoperative CT table and the operative table under anesthesia. In contrast, in the AR-portable navigation system, the FPP was determined using a gyro sensor that calculated the gravitational vectors, and changes in pelvic tilt showed no effect on RA. Additionally, in this system, RI was determined solely by bilateral ASIS registration, whereas adding the registration of the acetabular articular surface also affected the RI in the CT-based navigation system. These difference in the registration parameters could contribute to the accuracy of RI.

This study had some limitations. First, the use of CT for navigation preparation and postoperative evaluation has significant drawbacks, including radiation exposure and increased costs. Second, patients with extreme hip dislocations were excluded from this study. Using a CT-based navigation system, accurate cup placement has been reported even in cases of severe pelvic deformities, such as Crowe group IV^28^. Third, noise and metal artifact from post-THA CT scans might affect the measurement of RI and RA (Fig. 6). Fourth, this study did not include clinical results. Whether small errors in RI and RA would result in clinically important differences that patients could acknowledge in the long term would remain unknown. Several studies have questioned the utility of the Lewinnek safe zone^29–33^, as it may not always accurately predict the stability of THA. Determining the definitive target zone for cup placement is challenging due to the multifactorial nature of dislocation following THA^31^. Some researchers have proposed a functional safe zone based on hip and pelvic motion in the sagittal plane^33^. For instance, Tezuka et al.^33^ demonstrated that 14% of hips within the Lewinnek safe zone were outside this functional safe zone. Such findings highlight a potential reason for dislocation after THA, even in cases for which cup angles were considered “normal” according to traditional navigation techniques^29^. The placement of all cups within this so-called “safe zone” does not guarantee implant stability and longevity. Fifth, in this study, CT was performed in all cases to compare the accuracy of AR CT-based and portable AR-based navigation systems. If surgeons use the portable AR-based navigation system alone, preoperative CT is not needed. However, in this study, the possibility of reducing CT exposure was not proven. Finally, although the AR CT-based navigation system provides many functions, including reamer position, cup position, identifying muscles and blood vessels, further studies using cadavers are warranted to prove the usefulness of identifying blood vessels.

## Conclusion

The accuracy of cup insertion demonstrated no differences between the AR CT-based and portable AR-based navigation systems.

## Data Availability

The datasets generated during and/or analyzed during the current study are available from the corresponding author on reasonable request.
